# Beneficial effects of *Bacillus mojavensis* strain MTC-8 on plant growth, immunity and disease resistance against *Magnaporthe oryzae*

**DOI:** 10.3389/fmicb.2024.1422476

**Published:** 2024-06-12

**Authors:** Mu Ze, Feimin Ma, Jihong Zhang, Jichao Duan, Dingjin Feng, Yaoru Shen, Guanwei Chen, Xiaohong Hu, Ming Dong, Tuo Qi, Lijuan Zou

**Affiliations:** Ecological Security and Protection Key Laboratory of Sichuan Province, Mianyang Teachers' College, Mianyang, China

**Keywords:** *Bacillus mojavensis*, beneficial effects, plant growth, immunity, disease resistance, *Magnaporthe oryzae*

## Abstract

Rice blast, a prevalent and highly destructive rice disease that significantly impacts rice yield, is caused by the rice blast fungus. In the present study, a strain named MTC-8, identified as *Bacillus mojavensis*, was demonstrated has strong antagonistic activity against the rice blast fungus, *Rhizoctonia solani, Ustilaginoidea virens*, and *Bipolaria maydis*. The potential biocontrol agents were identified using ultra-performance liquid chromatography—tandem mass spectrometry (UPLC-MS/MS) analysis and chromatography. Further investigations elucidated the inhibitory mechanism of the isolated compound and demonstrated its ability to suppress spore germination, alter hyphal morphology, disrupt cell membrane integrity, and induce defense-related gene expression in rice. MTC-8 promoted plant growth and may lead to the development of a biocontrol agent that meets agricultural standards. Overall, the *Bacillus mojavensis* MTC-8 strain exerted beneficial effects on plant growth, immunity and disease resistance against rice blast fungus. In this study, we isolated and purified a bioactive substance from fermentation broth, and the results provide a foundation for the development and application of biopesticides. Elucidation of the inhibitory mechanism against rice blast fungus provides theoretical support for the identification of molecular targets. The successful development of a biocontrol agent lays the groundwork for its practical application in agriculture.

## 1 Introduction

Rice (*Oryza sativa* L.) is one of the most important cereal crops in the world, and it serves as a staple food for more than half of the global population (Chen et al., 2022; Saud et al., 2022). Rice plays a crucial strategic role in economies and societies, particularly in developing countries (Wang et al., 2016). However, diseases and pests have always constrained the high yield, stability, and quality of rice (Li et al., 2019; Nutan et al., 2020). Rice blast is caused by the filamentous fungus *Magnaporthe oryzae*, and it is one of the most serious threats to rice production worldwide (Dean et al., 2012). The efficacy of fungicidal control against pathogens is constrained by resistance issues, which supports investigations of biopesticides (Chakraborty et al., 2021). The use of antagonistic microorganisms and bioactive secondary metabolites is a sustainable approach for combatting blast fungi within the ecological framework and ensuring safety for humans and livestock (He et al., 2021; Lahlali et al., 2022); their use also aids in the mitigation of pest resistance by delaying the onset and progression of resistance mechanisms.

Bacteria are known for their widespread distribution, diverse species, high abundance, rapid multiplication, ease of cultivation, versatile mechanisms, and ability to establish dominant populations within plant ecosystems, but bacteria also play pivotal roles in plant disease control (Bonaterra et al., 2022). *Bacillus* spp., exemplified by *Bacillus thuringiensis*, produce spores that exhibit remarkable resilience to harsh environments and robust resistance to external stressors (Dobrzyński et al., 2023; Zhang et al., 2023). Several excellent Bacillus strains have been applied in production practices. The *Bacillus subtilis* strains GB03, MBl600, QST713, and FZB244 have been approved by the Environmental Protection Agency (EPA) for commercial production applications (Joshi and McSpadden Gardener, 2006; Kovács, 2019; Карпов et al., 2020). Biopesticide formulations of the *Bacillus subtilis* strain QST713 has been used for disease control of *Trichoderma aggressivum* f. *europaeum*, which is the main button mushroom green mold competitor that causes dysbiosis in weaned piglets (Pandin et al., 2018; Tsukahara et al., 2020). *Bacillus aryabhattai* (LSG3-7) and *Bacillus mojavensis* (LSG3-8) stimulate immune responses and improve antioxidant capacity in fish and have beneficial effects on the growth and disease resistance of *Rhynchocypris lagowskii* (Elsadek et al., 2023). Bacillus CB-R05 colonizes rice tissues, competes for nutrients with rice blast pathogens, secretes antibacterial compounds, inhibits pathogen growth, and triggers rice defense responses against blast invasion (Ji et al., 2014). These bacteria are widely distributed, have straightforward nutritional needs, proliferate rapidly, and maintain stable traits. Bacillus species are typically isolated from plants and soil and hold significant promise for agricultural applications.

Bacillus bacteria produce a diverse range of metabolites during growth, including various active substances, such as enzymes, proteins (e.g., chitinases and glucanases), bacteriocins, antibiotics, lipopeptides (e.g., lipopeptide antibiotics and peptide antibiotics), and other antimicrobial compounds (Raaijmakers et al., 2010; Zhao et al., 2018; Zhang et al., 2023). Biocontrol Bacillus strains trigger the plant's induced systemic resistance (ISR) signaling pathway to enhance the plant's ability to resist diseases (Pieterse et al., 2014; Samaniego-Gámez et al., 2023). The induction of ISR by biocontrol Bacillus primarily relies on the jasmonic acid (JA) and ethylene (ET) signaling pathways, but pathogen-induced plant systemic acquired resistance (SAR) primarily depends on the salicylic acid (SA) signaling pathway (Durrant and Dong, 2004; Klessig et al., 2018). ISR and SAR exhibit similar phenotypic features, including the induction of lignin formation, the accumulation of hydroxyproline-rich glycoproteins (HRGPs), the production of pathogenesis-related proteins (PR proteins), phenolic compound accumulation, and activation of host plant defense enzyme activities (Yi et al., 2013; Yadav et al., 2024).

In this study, we isolated a bacterium with strong antagonistic effects against rice blast fungus, MTC-8, which was identified as *Bacillus mogaveroi*. The fermentation broth of MTC-8 demonstrated excellent control efficacy, strong antibacterial activity, and stable production of secondary metabolites. The aim of the present study was topurify and separate the antibacterial secondary metabolites produced by MTC-8 and examine the mechanisms underlying its biocontrol effects to lay the foundation for the development and application of biopesticides. We investigated the impact of the active components on the germination of rice blast spores, pathogenicity, membrane permeability, and induction of defense gene expression in rice plants. The results provide initial insights into the mechanisms of its biocontrol effects. In the present study, we evaluated the plant growth-promoting effects of MTC-8 to promote its development as a highly effective biocontrol agent. Our study provides valuable insights into the isolation and screening of biocontrol bacteria, which contribute to the development of effective and eco-friendly agricultural practices.

## 2 Materials and methods

### 2.1 Isolation and identification of biocontrol bacterium

Pieces of roots, stems, and leaves from rice plants were washed with sterile water, followed by 75% ethyl for 1 min and rinsed with sterile distilled water 3 times, then disinfected in 5% sodium hypochlorite for 5 min and rinsed 6 times with ddH_2_O, these fragments were then placed in a sterile mortar and ground thoroughly, diluted proportionally and then 100 μL of each was spread on PDA medium at 25°C for 5 days. Sixteen purified strains were exhibited various degrees of inhibitory activities against *M. oryzae*, among these, the strain demonstrated the highest antagonistic activity against the rice blast fungus resulting in the conspicuous formation of an inhibitory zone during co-cultivation was designated as MTC-8 for subsequent research. The discovery of the biocontrol bacterium occurred serendipitously during the cultivation of *M. oryzae* in CM medium, which resulted in the conspicuous formation of an inhibitory zone. 16S RNA was amplified using specific primers (27F: AGA GTTTGATCMTGGCTCAG; 1492R: CGGTTACCTTGTTACGACTT) (Wolf et al., 2014), and the resulting sequence was deposited in GenBank as PP627040. Phylogenetic analysis was performed using the neighbor-joining (NJ) method in MEGA 7.0 software (RRID:SCR_011920) (https://www.megasoftware.net/) (Kumar et al., 2016).

### 2.2 Antifungal activity and pathogen infection experiments

Pathogenic plant fungi, such as *Ustilaginoidea virens, Magnaporthe oryzae, Rhizoctonia solani*, and *Bipolaris maydis*, were collected and maintained in our laboratory. The *M. oryzae* strain Guy11 was used as the indicator strain. The *M. oryzae* Guy11 strain was cultured on CM at 28°C for 5 days, and Guy11 spores were cultivated in tomato and oat culture media at 28°C for 8 days.

Guy11 was inoculated onto a CM plate and alternately cultured in darkness and light at 28°C for 7–11 days. Sterile distilled water (2 mL) was added to each well-developed rice blast plate. The colony surface was gently scraped with the tip of a pipette, and any impurities or mycelia were removed using a Miracloth filter cloth. The mixture was centrifuged at 8,000 r/min for 3 min, and the supernatant was discarded. The process was repeated twice.

The concentration of conidia was adjusted to approximately 1x10^5^ conidia/mL using a hemocytometer under a microscope via the addition of an appropriate volume of sterile distilled water. The active substances F1, F2, F3, F4, and sterile distilled water were separately introduced into the spore solution at the adjusted concentrations.

A clean, hydrophobic slide was used to absorb 30 μL of spore droplets, which were placed in a humid petri dish for continuous cultivation at 28°C. At intervals of 0, 4, 12, 24, 36, and 48 h during the cultivation process, cover slides were placed, sealed with Vaseline, and assembled into plates. The germination of conidia and the formation of appressoria were observed under a microscope.

Once rice plants reached the two-leaf stage, 5–7 cm leaves were carefully excised from the uppermost leaves for fungal inoculation. Using the tip of a 10 μL pipette, a wound was gently created on the rice leaf surface, which was then immersed in a 6-BA solution (1 mg/L, pH 7.0). The maintenance of consistent pressure was crucial during this process. The conidia produced by *M. oryzae* were collected from CM media, and any impurities or mycelia were removed using a Miracloth filter cloth. The spore concentration was adjusted to 2 × 10^5^ conidia/mL using a hemocytometer. The active substances F1, F2, F3, F4, and sterile distilled water were combined with the spore suspension, and 5 μL of the mixture was carefully applied to each wound. After inoculation, the infected leaves were placed in a controlled environment chamber under dark conditions for 12 h. The chambers were shifted to a 12-h light/12-h dark cycle for 5–7 days to monitor wound development, capture images, and measure lesion sizes. The number of lesions was recorded for each treatment. Lesion lengths were documented and quantified using ImageJ (RRID: SCR_003070) software, and observations of spores and mycelia were performed using an optical microscope (Olympus, Japan).

### 2.3 Thin layer chromatography, column chromatography and LC–MS

For column chromatography, 200-mesh silica gel was introduced into the column through a funnel. To ensure continuous, uniform, and compact loading of the silica gel, a rubber mallet was used to gently tap the column. The silica gel was added to approximately three-quarters of the column's height. The lower end piston was opened, and air pressure was applied using an air pump to compress the column bed volume. A layer of quartz sand, approximately 1- to 2-cm thick, was carefully added on top of the column.

The prepared eluent was poured into the column, and pressure was applied with an air pump to thoroughly wet the silica gel column. Any bubbles in the column were expelled, and the eluent liquid surface was maintained approximately 2 to 3 cm away from the column surface.

The concentrated MTC-8 ethyl acetate extract for separation was dissolved in approximately 1 mL of methanol to create a small-volume, high-concentration sample solution. Using a plastic dropper, the sample solution was delicately added onto the column surface to avoid disruption of the column surface and maintain the silica surface.

The sample was pressed from the quartz sand layer down to the silica column layer, approximately 2 cm deep. The column was washed with the selected eluent (n-hexane:ethyl acetate = 15:1), and an eluent flow rate of 1 to 2 drops/second was maintained, with continuous addition of eluent at the top of the column. Samples were collected in 20-mL test tubes, and each tube contained one sample. Each sample was examined using thin-layer chromatography (TLC), and samples containing the same components were combined. The separated sample was obtained using rotary evaporation and concentration.

To identify the specialized metabolites generated by MTC-8, the fermentation filtrate broths were collected and preserved at −80°C before being subjected to ultra-performance liquid chromatography (UPLC)–tandem mass spectrometry (UPLC-MS/MS) analysis at MetWare Biotechnology Co., Ltd.

### 2.4 qRT-PCR assay

LTH seeds were immersed in a 3% H_2_O_2_ solution for 12 h then rinsed with water until they appeared white. These treated rice seeds were sown in a 96-well hydroponic container and placed in a light-controlled growth chamber for 28 days.

A solution of 10 mL of the active substance solution F3 and water was prepared and sprayed onto the rice leaves using a watering can. Rice leaves were harvested at 0, 12, and 24 h after inoculation.

Extraction of Total RNA from Rice: Approximately 80–100 mg of rice leaves from each treatment was carefully excised and placed in RNase-free EP tubes. The leaves were pulverized to a fine powder by incorporating liquid nitrogen. The RNA extraction process involved the addition of 1,000 μL of TRIzol extraction solution, followed by reverse transcription of RNA using TaKaRa's reverse transcription kit. After RNA was extracted, quantitative real-time polymerase chain reactions (qRT-PCR) were conducted with specific primers targeting *OsMAS, OsPR1a, OsPR10* (pathogenesis-related genes, PRs), and other defense-related genes (genes related to diterpenoid synthesis, *OsKS4*), the sequences of these specific primers were displayed on Supplementary Table S2. The relative gene expression levels were normalized by comparison with the expression of ubiquitin 5 (*OsUbq5*) and assessed using the 2^−Δ*ΔCT*^ method (Maren et al., 2023; Shen et al., 2024). The data are presented as means ± standard deviation (SD).

## 3 Results

### 3.1 Antagonistic activity of the biocontrol bacterium *Bacillus mojavensis* MTC-8 against *M. oryzae*

**Figure 1:**
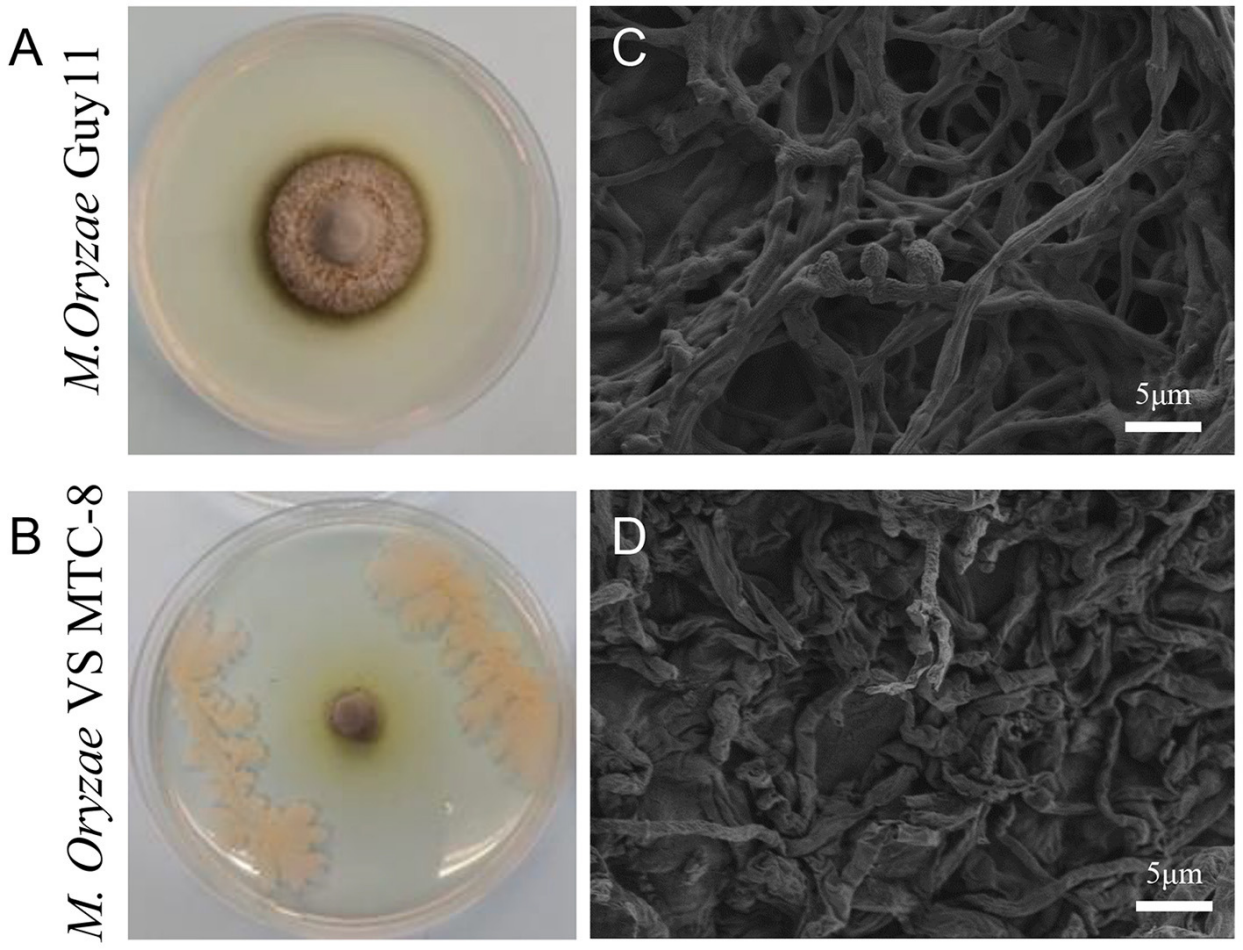
Antagonistic activity of the biocontrol bacterium *Bacillus mojavensis* strain MTC-8 against *the Magnaporthe oryzae* strain Guy11. **(A)** The mycelial morphology of *M. oryzae* strain Guy11. **(B)** MTC-8 was inoculated into Guy11 colonies, and the colonies were photographed. **(C)** The ultrastructure of Guy11 in the control group; scale bar = 5 μm. **(D)** The ultrastructure of Guy11 after co-cultivation with the biocontrol bacterium *Bacillus mojavensis* strain MTC-8; scale bar = 5 μm. The rice blast fungus Guy11 was cultured on CM media at 28°C for 5 days, and ultrastructure was examined using scanning electron microscopy (SEM).

In a rice blast disease nursery located in Wenjiang (103°83′ E, 30°70′ N), Sichuan province, China, rice plants in a specific plot exhibited resistance to rice blast disease. Consequently, the roots, stems, and leaves of these rice plants were collected and brough back to the laboratory for pathogen isolation. Antagonistic strains were screened in endophytic and epiphytic bacteria of rice, and 16 strains exhibited various degrees of inhibitory activity against *M. oryzae*. The strain that demonstrated the highest antagonistic activity against the rice blast fungus during co-cultivation was designated MTC-8 for subsequent research (Figures 1A, B). Mycelial morphology was examined using scanning electron microscopy (SEM). The ultrastructure of the rice blast fungus in the control group was intact, with smooth hyphal cell walls, a normal cell membrane structure, an undivided cytoplasmic wall, and normal conidia formation. After confrontation, *M. oryzae* exhibited abnormalities in hyphal morphology, protoplasm condensation, inhibition of conidial germination, and hyphal tip rupture (Figures 1C, D).

### 3.2 Identification and characteristics of MTC-8

**Figure 2:**
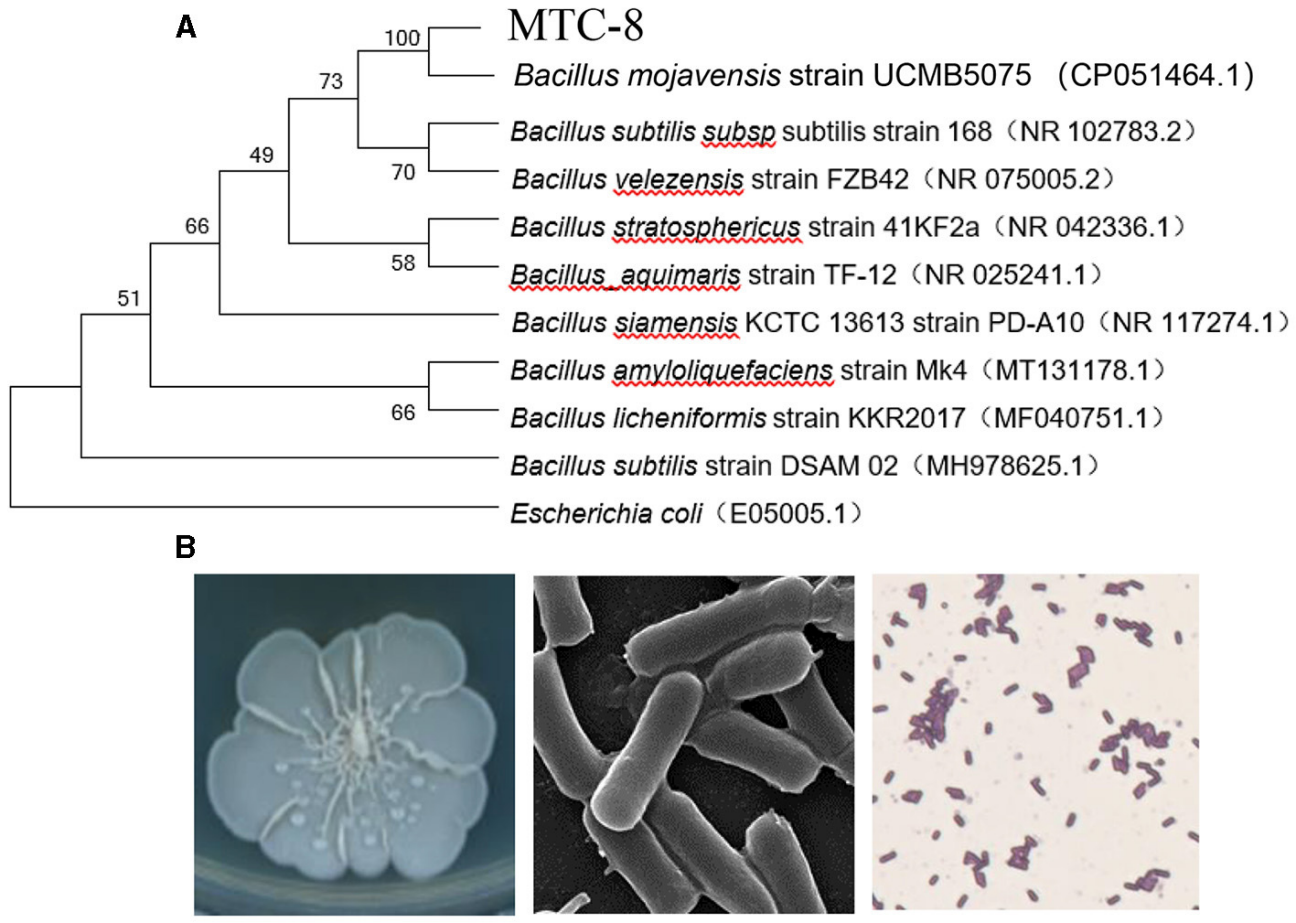
Identification and characteristics of the *Bacillus mojavensis* strain MTC-8. **(A)** A phylogenetic tree was constructed based on the complete 16S rRNA gene sequences of strain MTC-8 and other strains retrieved from GenBank. The tree was constructed by the neighbor-joining (NJ) algorithm, and 1,000 bootstrap replicates were performed by MEGA 7.0 software. The bootstrap value (%) is indicated proximal to the nodes. **(B)** The colony morphology, ultrastructure under the electron microscope and bacterial staining of the *Bacillus mojavensis* strain MTC-8.

**Figure 3:**
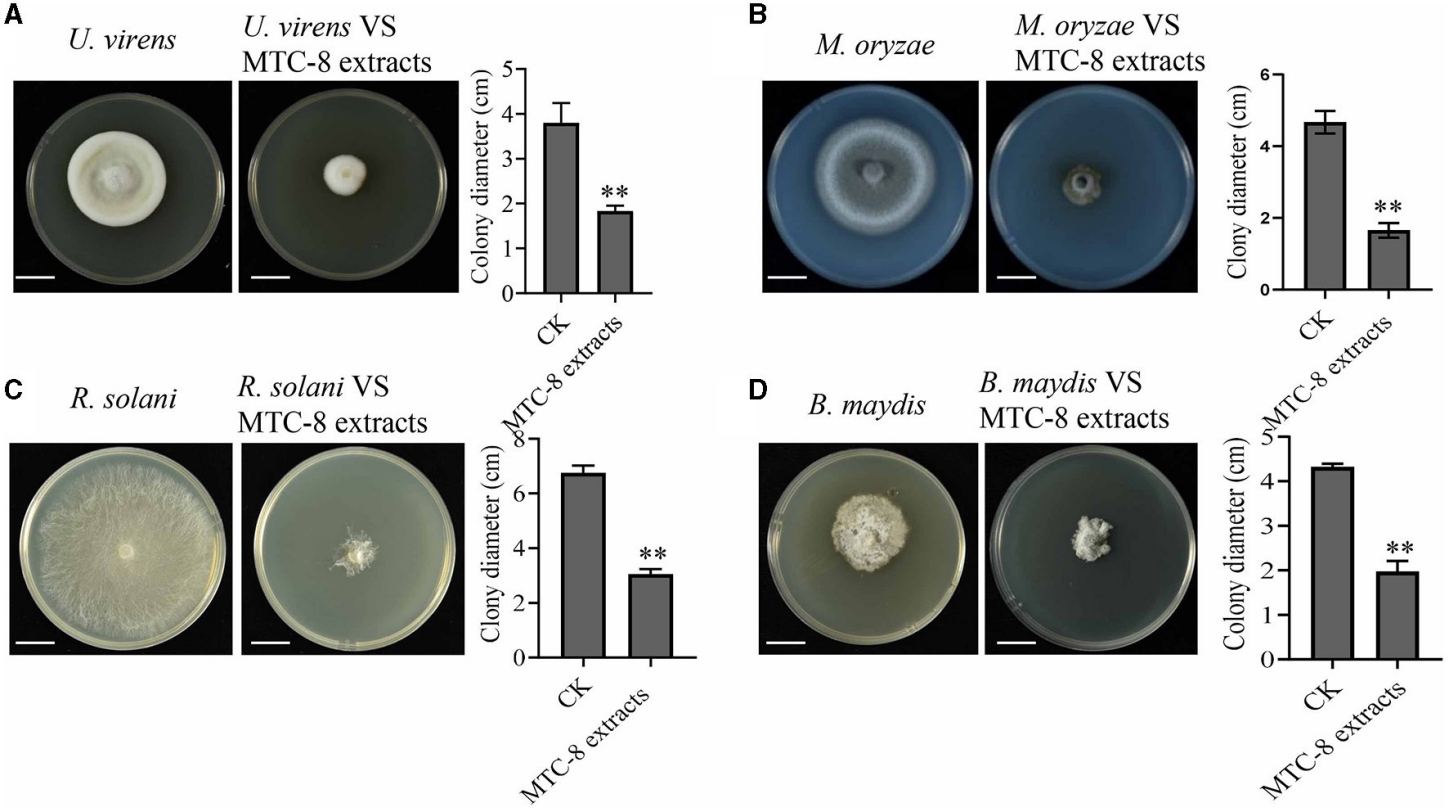
The antimicrobial spectrum of MTC-8 and its extract against phytopathogenic fungi. The mycelial morphology and colony diameters of the plant pathogenic fungi *Ustilaginoidea virens* **(A)**, *M. oryzae* **(B)**, *Rhizoctonia solani* **(C)**, and *Bipolaria maydis* **(D)**, which were cultured on drug plates supplemented with MTC-8 extracts. The MTC-8 extracts were grown in liquid PDB medium and added to the drug plates, and the drug plates without the MTC-8 extract broth were used as controls (CKs). Scale bar = 2 cm. The values are the means ± SDs. Asterisks denote a significant difference from the CK as determined by Student's *t*-test (^*^0.01 < *p* < 0.05; ^**^p < 0.01).

To identify MTC-8, we amplified 16S ribosomal RNA genes. As shown in Figure 2A, a BLAST analysis of the complete 16S rRNA gene sequences revealed that the MTC-8 strain was closely related to *Bacillus mojavensis* strain UCMB5075 (CP051464.1) with 100% similarity. A phylogenetic tree was constructed using MEGA7.0 software. MTC-8 was grouped with *B. mojavensis* strain UCMB5075 with a high bootstrap value (1,000; Figure 2A). Overall, MTC-8 was recognized as a member of the *B. mojavensis* family. The MTC-8 strain appeared milky white with irregular edges on the PDA plate, with a wrinkled biofilm on the colony surface. Under the electron microscope, it appeared rod-shaped (Figure 2B). Bacterial staining experiments identified MTC-8 as a Gram-positive bacterium with spores (Figure 2B). The growth characteristics of MTC-8 cultivated in PDB liquid media were analyzed, and the OD values were measured. As shown in Supplementary Figure S1, MTC-8 entered the logarithmic phase after 12 h, reached a plateau phase after 48 h, then gradually declined. Therefore, the optimal fermentation time for MTC-8 should be 48 h. To further determine the antimicrobial spectrum of MTC-8, we performed drug plate experiments on *Rhizoctonia solani, Ustilaginoidea virens, Bipolaria maydis*, and *M. oryzae*. Figure 3 shows that the fermentation broth of MTC-8 effectively inhibited the growth of plant pathogenic fungi. With the addition of fermentation broth to the drug plate, the colony diameters of *M. oryzae, U. virens, R. solani*, and *B. maydis* were 1.65 cm, 1.97 cm, 1.83 cm, and 3.05 cm, respectively. Compared to the control, all of the other strains were significantly inhibited, which indicated that the fermentation broth of MTC-8 had an excellent antagonistic effect on different plant pathogens (Figure 3).

### 3.3 Assay of the effect of MTC-8 extract on controlling rice blast

**Figure 4:**
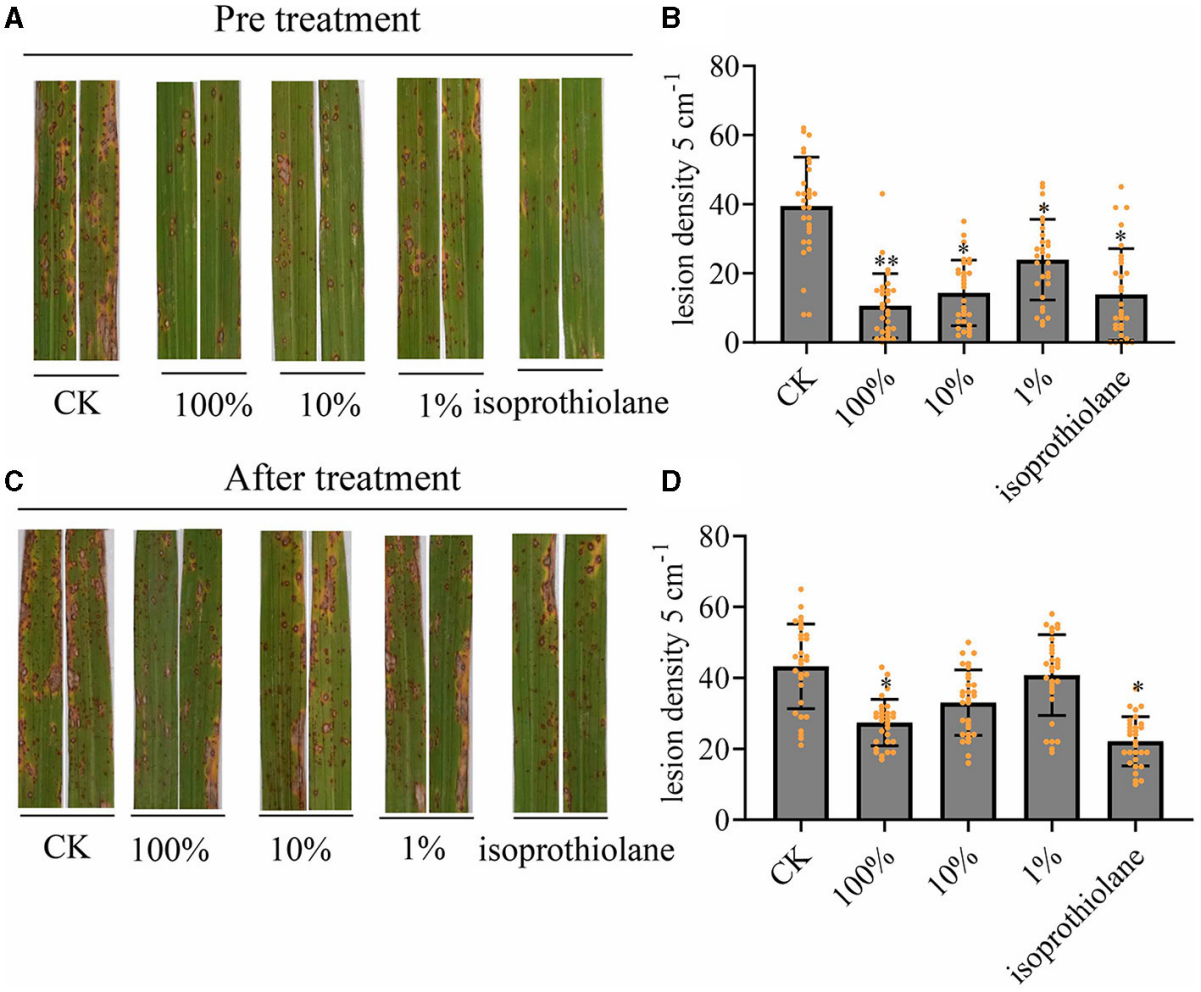
*In vivo* effect of MTC-8 extract on rice leaves and disease incidence caused by *M. oryzae*. **(A, B)** Lesion symptoms and blast disease lesion density after the preventive treatment, which involved spraying the fermentation broth then *M. oryzae* spores 24 h later on field-grown rice plants. Rice leaves were sprayed with 0 (CK), 1, 10, or 100% MTC-8 extract, or the chemical fungicide isoprothiolane 24 h before inoculation with Guy11 spores. **(C, D)** Lesion symptoms and blast disease lesion density after the therapeutic treatment, which involved spraying rice blast spores followed by the fermentation broth 24 h later. Rice leaves were sprayed with 0 (CK), 1, 10, or 100% MTC-8 extract, or the chemical fungicide isoprothiolane 24 h after inoculation with Guy11 spores. Blast disease lesion density was quantified in infected leaf segments (5 cm in length) 5 days post-infection. The values are the means ± SDs. Asterisks denote a significant difference from the CK as determined by Student's *t*-test (^*^0.01 < *p* < 0.05; ^**^p < 0.01). *n* = 30 independent leaves in **(B, D)**.

We sprayed the fermentation broth of MTC-8 onto rice plants growing in the field and performed preventive and therapeutic treatments with the fermentation broth. The preventive treatment involved spraying the fermentation broth first then the *M. oryzae* spores 24 h later. The therapeutic treatment involved spraying rice blast spores then the fermentation broth 24 h later. By counting the number of rice blast lesions on rice leaves after fermentation broth treatment, we examined the preventive and therapeutic effects of the biocontrol agent MTC-8 against rice blast disease. The results of lesion counting for each treatment group are shown in Figure 4. The number of lesions after preventive treatment with MTC-8 fermentation broth was significantly lower than the control group (Figures 4A, B). Increased concentrations of the fermentation broth decreased the number of lesions. The number of lesions after treatment with the undiluted fermentation broth was comparable to the chemical fungicide (Isoprothiolane) treatment, which indicated that the MTC-8 fermentation broth also has a good preventive effect against rice blast disease in the field. The therapeutic treatment also showed good results, but the preventive effect was significantly more pronounced (Figures 4C, D).

### 3.4 Active antimicrobial substances analysis

**Figure 5:**
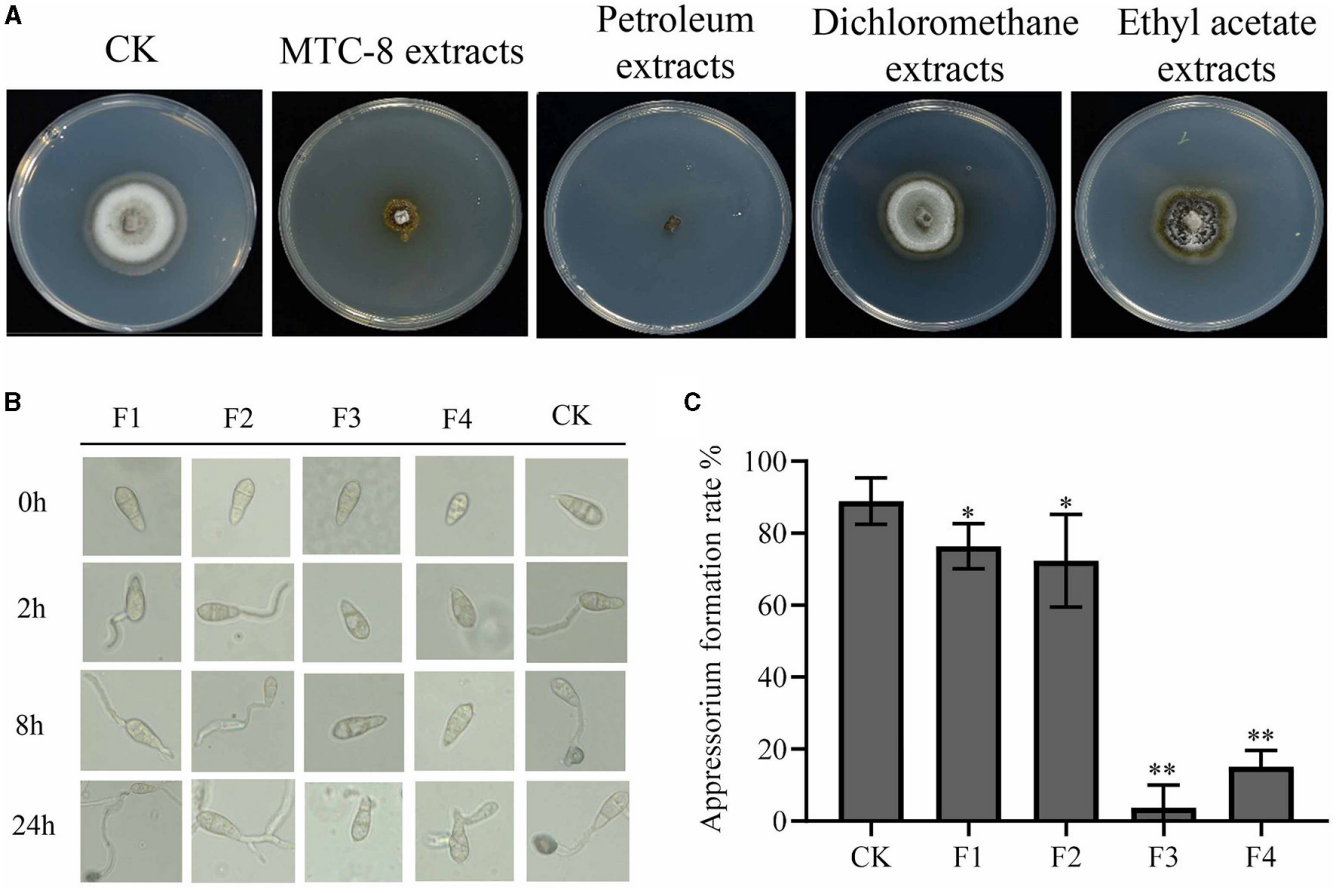
Active antimicrobial substance analysis of strain MTC-8. **(A)** The mycelial morphology of Guy11 cultured on CM containing MTC-8 extracts, petroleum extracts, dichloromethane extracts, and ethyl acetate extracts. The germination of conidia and the formation of appressoria **(B)** and the appressorium formation rate **(C)** of Guy11, which was inoculated on hydrophobic cover slips, and appressorium formation were observed at 0, 2, 8, and 24 hours after the Guy11 conidia suspensions were treated with F1, F2, F3, F4 and non-treated Guy11 conidia suspensions as a control (CK). The values are the means ± SDs. Asterisks denote a significant difference from the CK as determined by Student's *t*-test (^*^0.01 < *p* < 0.05; ^**^p < 0.01).

We investigated the potentially effective antifungal agents of MTC-8 that suppressed rice blast. MTC-8 fermentation broth was extracted with water, petroleum ether, dichloromethane, and ethyl acetate and concentrated using a rotary evaporator. The obtained substances were further tested for activity on drug plates. The antimicrobial activity of the petroleum ether extract was the strongest (Figure 5A), followed by the aqueous phase extract. The antifungal activities of the dichloromethane and ethyl acetate extracts were relatively poor, which indicated that the active substances were more soluble in petroleum ether. Therefore, petroleum ether was chosen as the extraction solvent for subsequent experiments. Thin-layer chromatography separation experiments showed that when the mobile phase was a mixture of n-hexane and ethyl acetate at a ratio of 15:1, the active substances obtained from the extraction could be separated. This ratio was used for preparing the mobile phase in subsequent experiments. The extract was further separated using column chromatography with thin-layer chromatography (Supplementary Figure S2). The eluents containing different components separated by the silica gel column were spotted and detected using thin-layer chromatography. Samples containing the same components were combined, which resulted in the separation of 4 components, named F1, F2, F3, and F4. The MTC-8 extracts were mixed with 4 × 10^5^ conidia/mL Guy11 spore suspensions in equal volumes and incubated on hydrophobic slides (Figure 5B).

The spores in the control group began to germinate and produce germ tubes after 2 h. By 8 h, the germ tubes further elongated and formed attachment cells at the other end. At 24 h, the attachment cell formation rate reached 88.91%, as shown in Figure 5B. Compared to the control group, germ tubes were produced 2 h after treatment with F1 and F2, but the formation of attachment cells was slightly delayed at 8 h. By 24 h, attachment cell structures were observed in F1 and F2, with attachment cell formation rates of 76.40% and 72.37%, respectively. After treatment with F3 and F4, the F3-treated group did not germinate germ tubes at 2 h, and no germ tubes or attachment cell structures were formed at 8 or 24 h, which resulted in an attachment cell formation rate of only 3.67%. The F4-treated group did not germinate germ tubes at 2 h, and no germ tubes or attachment cell structures were formed at 8 h. After 24 h, germ tube germination occurred, but attachment cells were not formed, with an attachment cell formation rate of 15.09% (Figure 5C). These results indicate that F1 and F2 only partially delayed the formation of attachment cells but did not inhibit the production of attachment cell structures. In contrast, F3 strongly inhibited the germination of spore germ tubes and the formation of attachment cells, with only a few attachment cells formed within 24 h. F4 was considered the control group because it may contain a few active substances. Therefore, these results indicate that active substances are present in F3.

We performed LC–MS analysis of the F3 extract to further investigate the biocontrol agents present in the MTC-8 strain extract (Supplementary Figure S3). Comparative LC–MS analysis provided valuable insights into the biocontrol agents within the MTC-8 strain extract. Total ion chromatography (TIC) revealed that the molecular weights of the metabolites were <3,000 Da, and approximately 9 substances exhibited high abundance. Subsequent substance identification relied on the secondary mass spectrometry data obtained from online and local databases. The identified substances and their respective proportions in F3 are detailed in Supplementary Table S1. N-Phenyl-2-naphthylamine, phthalic anhydride, dibutyl phthalate, hexadecanamide, 2-deoxyhexopyranose, and isopropylmalic acid were among the substances detected, which may contribute to the antifungal activity. However, further analysis is warranted to elucidate their specific antifungal properties.

### 3.5 Plant growth-promoting activity of MTC-8

**Figure 6:**
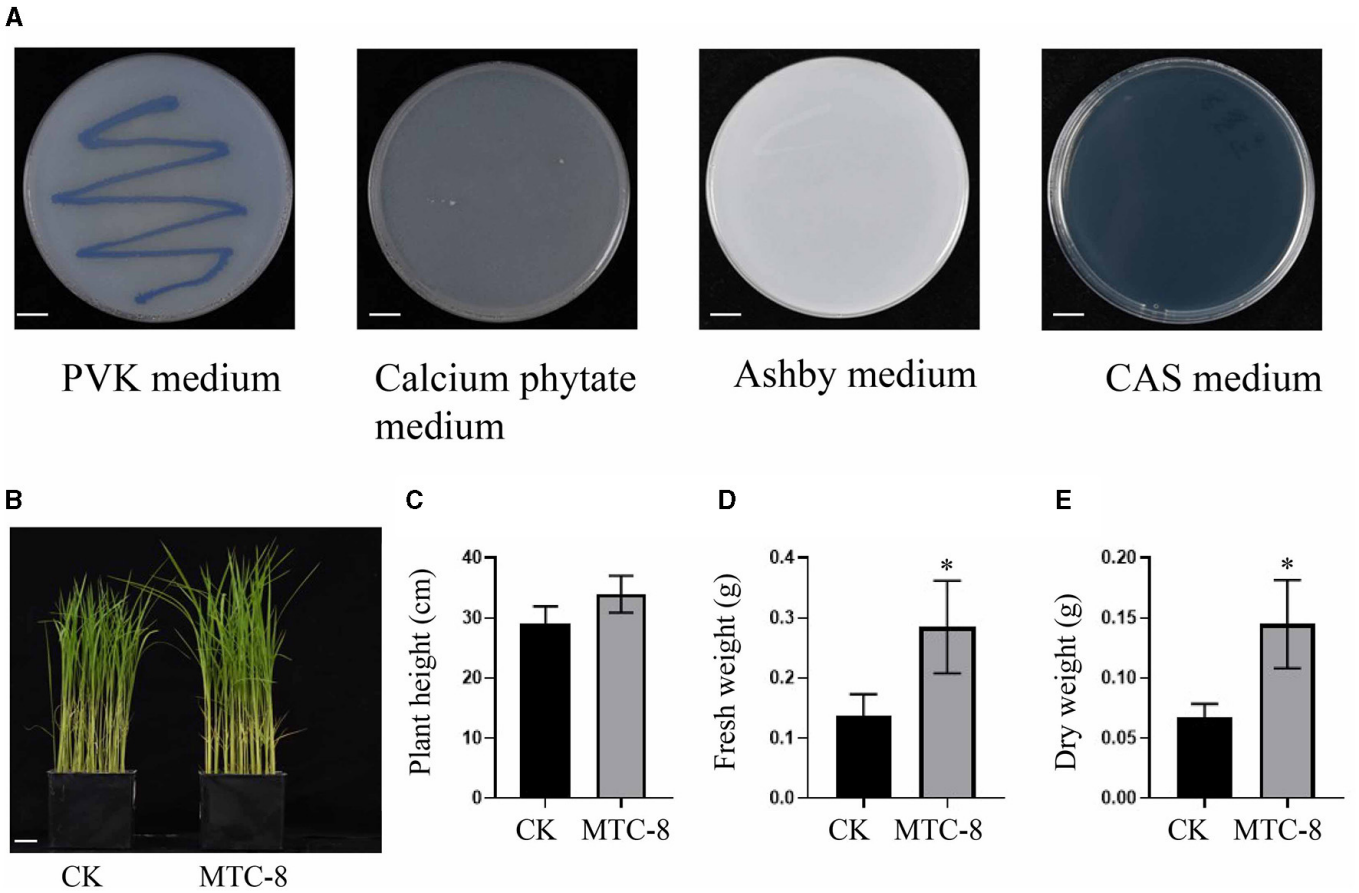
The plant growth-promoting and immunity elicitor potential of MTC-8. **(A)** Colony characteristics of MTC-8 cells cultured on PVK, calcium phytate, Ashby, and CAS media; PVK media for phosphorus solubilization ability; calcium phytate media for organic phosphorus solubilization ability; Ashby media for nitrogen fixation ability; and CAS media for iron carrier screening. Phenotype **(B)**, plant height **(C)**, fresh weight **(D)**, and dry weight **(E)** of TP309 rice plants after inoculation with MTC-8 bacterial liquid; pure PDB liquid was used as a control. The values are the means ± SDs. Asterisks denote a significant difference from the CK as determined by Student's *t*-test (^*^0.01 < *p* < 0.05). *n* = 3 in **(C–E)**. Scale bar = 1 cm in **(A)**, 2 cm in **(B)**.

In this study, we investigated the plant growth-promoting and immunity elicitor potential of MTC-8. We used PVK plates to assess its phosphorus solubilization ability and calcium phytate media to test organic phosphorus solubilization ability. Strains capable of growing on Ashby plates were evaluated for their nitrogen-fixing ability. The production of iron carriers was examined using CAS media. MTC-8 demonstrated an ability to solubilize inorganic phosphorus by forming solubilization zones on PVK plates; however, it did not exhibit organic phosphorus solubilization ability because it failed to grow on calcium phytate plates. MTC-8 exhibited nitrogen-fixing ability by growing normally on Ashby plates. Notably, no orange halo was observed around MTC-8 colonies post-inoculation, which indicated the absence of iron carrier production ability (Figure 6A).

We also inoculated MTC-8 bacterial suspension onto TP309 rice plants to evaluate its impact on plant growth. The results indicated that rice plant growth was not negatively affected after MTC-8 bacterial inoculation, but a growth-promoting effect was observed (Figures 6B, C). Statistical analysis of plant height, fresh weight, and dry weight further supported these findings. The plant height of the treatment group (33.98 cm) exceeded the control group (29.17 cm), and similar trends were observed for fresh weight (0.28 g vs. 0.14 g) and dry weight (0.14 g vs. 0.07 g) (Figures 6C–E). These results collectively suggest that MTC-8 has growth-promoting abilities. The application of MTC-8 fermentation broth on 2-week-old rice leaves did not significantly impact leaf growth (Figure 6B) but did significantly increase the expression of *OsMAS, OsPR1a, OsPR10* (pathogenesis-related genes, PRs), and *OsKS4* (genes related to diterpenoid synthesis) after 12 h of treatment (Supplementary Figure S4, Supplementary Table S2).

## 4 Discussion

Rice blast poses a great threat to rice production (Dean et al., 2012; Li et al., 2019). Fungicidal control is limited due to pathogen resistance, which necessitates the investigation of biopesticides (Zhang et al., 2020, 2023; He et al., 2021). Microorganisms, including bacteria, fungi, viruses, and actinomycetes, have been extensively researched and used for plant disease control (Bonaterra et al., 2022; Boro et al., 2022); they hinder pathogen growth via diverse mechanisms, including competition, hyperparasitism, antimicrobial secretion, resistance induction, and growth stimulation (del Carmen Orozco-Mosqueda et al., 2018; Torres-Rodriguez et al., 2022). For example, endophytic bacteria bolster host plant resistance and evoke systemic plant defenses, and low-virulence fungi and viruses have emerged as valuable biological control agents due to their subdued virulence traits (Yu et al., 2013; Umar et al., 2021; Dai et al., 2024). Although biological control technology has made significant progress, it faces some challenges, such as the lack of highly efficient biocontrol strains, poor colonization ability of biocontrol agents, and weak resistance of biocontrol agents. Our study supports the marked effectiveness of the MTC-8 strain in suppressing rice blast fungus growth and evaluated its efficacy in combatting fungal diseases in crops, with a particular focus on rice blast disease control and its potential for enhancing rice growth. This research provides valuable insights into the beneficial role of MTC-8 in rice disease management and emphasizes its significance in the field of rice pathogen control.

The transmission of rice blast disease is facilitated by the asexual reproduction of spores. Because lesions on the leaves reach a certain size, the mycelia within them undergo differentiation to generate conidiophores, which generate to conidia. These conidiospores reinfect the plant to initiate a new cycle of asexual reproduction (Sesma and Osbourn, 2004; Ryder et al., 2019). The MTC-8 fermentation broth demonstrated strong preventive efficacy against rice blast disease under field conditions. The therapeutic treatment yielded positive outcomes, but the preventive effect was notably more pronounced. MTC-8 exerts its biocontrol effect against rice blast disease by colonizing the surface of rice leaves and occupying ecological niches. The infection process of rice blast fungus involves six key stages: conidiospore attachment to the host epidermis, germination leading to germ tube formation, development and maturation of appressoria, penetration and invasion, differentiation and elongation of invasive hyphae, sporulation, and subsequent reinfection (Hamer and Talbott, 1998; Ryder and Talbot, 2015). MTC-8 inhibited the growth of mycelia of plant pathogenic fungi, such as *M. oryzae, U. virens, R. solani*, and *B. maydis*. According to the plate confrontation test, *M. oryzae* exhibited anomalies in hyphal structure, protoplasm condensation, suppression of conidial germination, and rupture of hyphal tips. The secondary metabolites of MTC-8 inhibited the germination of rice blast fungus spores and the formation of appressoria, which led to abnormal germination of some spores and decreased their infectivity.

The biocontrol mechanisms of plant pathogens by *Bacillus* spp. include antagonism, competition, and induced systemic resistance (ISR) via the synthesis of secondary metabolites, which directly inhibit plant pathogens (Yi et al., 2013; Pieterse et al., 2014; del Carmen Orozco-Mosqueda et al., 2018; Samaniego-Gámez et al., 2023). *Bacillus* spp. release antagonistic metabolites, such as antimicrobial peptides, polyketides, lytic enzymes, and volatile organic compounds (VOCs), which directly suppress plant pathogens (Zhao et al., 2018; Boro et al., 2022; Zhang et al., 2023). MTC-8 produced benzene and substituted derivatives, carboxylic acids and derivatives, fatty acyls, benzothiazoles, indoles and derivatives, organic phosphoric acids and derivatives, organonitrogen compounds, and prenol lipids. The relative percentages of substances in sample F3, including N-phenyl-2-naphthylamine, phthalic anhydride, dibutyl phthalate, hexadecanamide 2-deoxyhexopyranose, and isopropylmalic acid, were the highest. N-phenyl-2-naphthylamine (P2NA) is a plant secondary metabolite that is commonly used as an antioxidant. Phthalic anhydride and its derivatives have antibacterial and antifungal activity (Khdera et al., 2022; Sousa et al., 2024). Only a small fraction of secondary metabolites have been identified due to limitations in chemical database retrieval. We also used AntiSMASH to identify secondary metabolite regions and showed that MTC-8 produced bacillibactin, pulcherriminic acid, RiPP, thiopeptide, fengycin, and thermoactinoamide A (data not shown). MTC-8 generated beneficial agents with direct biocontrol activity and altered the microbiome to boost plant disease suppression.

Some biocontrol bacteria directly and indirectly enhance plant growth and defenses against pathogen intrusion via several actions, such as antibiotic production, induced systemic resistance (ISR), and competition for nutrients and habitats (Lugtenberg and Kamilova, 2009; Vejan et al., 2016; De Andrade et al., 2023). MTC-8 solubilized inorganic phosphorus and demonstrated nitrogen-fixing capabilities. MTC-8 promoted the growth of rice plants during the seedling stage after only 2 weeks of treatment while significantly triggering the expression of plant immune-related genes (OsMAS, OsPR1a, OsPR10, and OsKS4). *Streptomyces alfalfae* XN-04 exhibits significant inhibitory activity against *Fusarium oxysporum* f. sp. *vasinfectum* (*Fov*). XN-04 also combats *Fov* and enhances plant growth via the secretion of hydrolytic enzymes, indoleacetic acid and phosphate solubilization (Chen et al., 2021). *Bacillus mojavensis* I4 enhances salt stress tolerance in durum wheat by improving physiological parameters, reducing membrane damage, increasing antioxidant enzyme activities, and enhancing stress adaptation (Ghazala et al., 2023). Although the antiSMASH results indicated that MTC-8 produced bacillibactin, its inability to grow on CAS media suggests that MTC-8 lacks the ability to produce iron carriers. The biocontrol mechanisms of MTC-8 include regulation of hormonal and nutritional balance, induction of resistance against plant pathogens, and facilitation of nutrient solubilization to enhance plant uptake. However, the beneficial effects of MTC-8 on plant growth promotion await further exploration.

## 5 Conclusion

In this study, we examined the beneficial effects of the *Bacillus mojavensis* MTC-8 strain on plant growth, immunity, and disease resistance against the rice blast fungus. The results obtained in this study highlight the strong antagonistic activity of MTC-8 against various plant pathogens and its ability to promote plant growth. LC–MS and chromatography identified bioactive substances, and the inhibitory mechanism of these compounds was elucidated. MTC-8 affected spore germination, hyphal morphology, cell membrane integrity, and defense-related gene expression in rice. Our research supports the potential of MTC-8 as a biocontrol agent that can meet agricultural standards, which provides a foundation for the development and application of biopesticides. Overall, we identified MTC-8 as *Bacillus mojavensis*, demonstrated its antagonistic activity against multiple plant pathogens, isolated and purified its bioactive substances and elucidated its mechanism of action against the rice blast fungus. Our results lay the groundwork for the development and application of biopesticides in agriculture.

## Data availability statement

The original contributions presented in the study are included in the article/Supplementary material, further inquiries can be directed to the corresponding authors.

## Author contributions

MZ: Funding acquisition, Writing – original draft, Writing – review & editing, Data curation, Investigation, Visualization. FM: Investigation, Visualization, Writing – original draft. JZ: Writing – review & editing. JD: Investigation, Visualization, Writing – review & editing. DF: Investigation, Visualization, Writing – original draft. YS: Writing – review & editing. GC: Investigation, Visualization, Writing – review & editing. XH: Funding acquisition, Supervision, Writing – review & editing. MD: Funding acquisition, Supervision, Writing – review & editing. TQ: Conceptualization, Data curation, Funding acquisition, Project administration, Supervision, Writing – original draft, Writing – review & editing. LZ: Conceptualization, Data curation, Formal analysis, Funding acquisition, Project administration, Supervision, Writing – original draft, Writing – review & editing.
