# Supplementary material for: Beneficial effects of Bacillus mojavensis strain MTC-8 on plant growth, immunity and disease resistance against Magnaporthe oryzae
Source: Front Microbiol. 2024 Jun 12;15:1422476. doi: 10.3389/fmicb.2024.1422476 (PMC11199545; doi:10.3389/fmicb.2024.1422476)
Supplement: Supplementary file 2 [file Data_Sheet_2.docx]

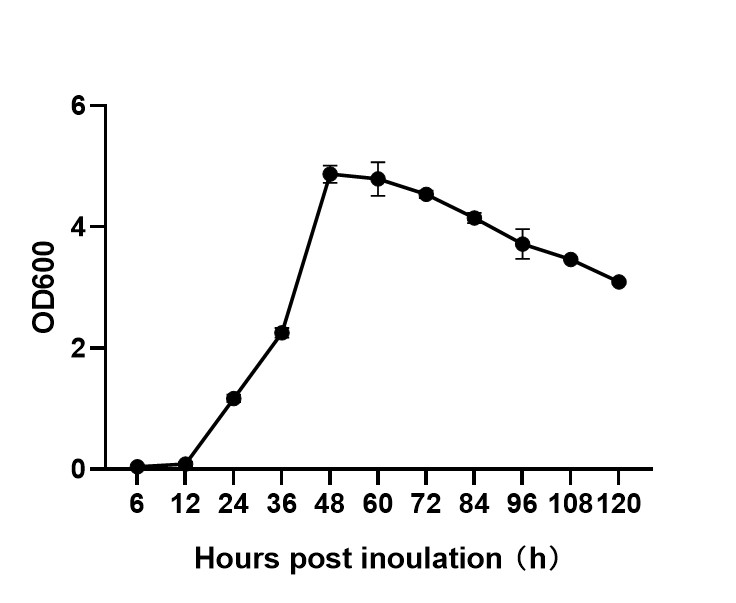


**Supplementary Figure 1.** The growth characteristics analyzing of MTC-8. Y-axis represented OD value, X-axis represented hours post inoculation MTC-8 cultivated strain using PDB liquid medium.


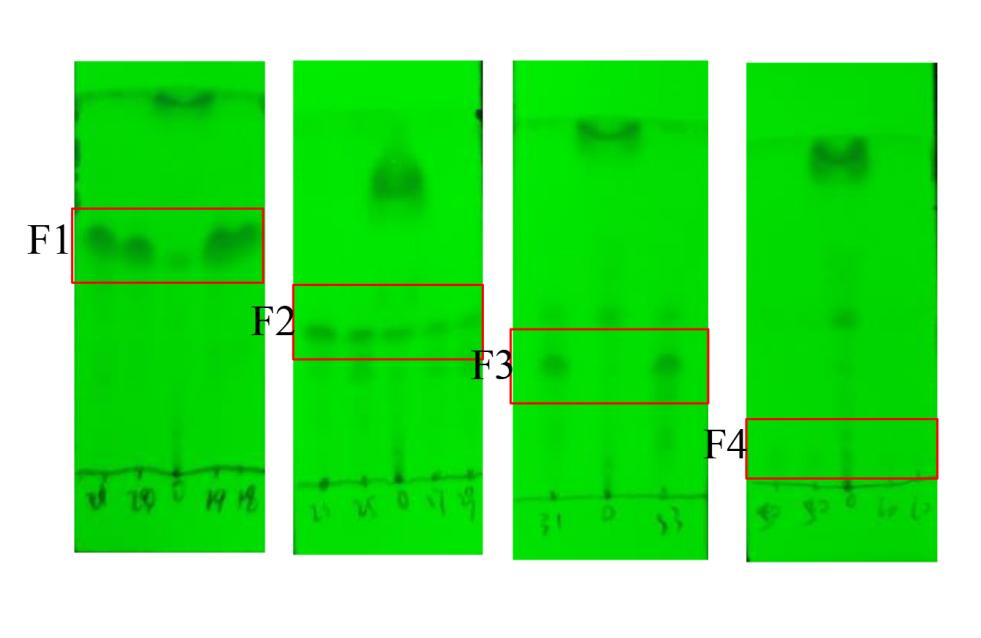


**Supplementary Figure 2.** Four segments of ethyl acetate extract separated from strain MTC-8 using column chromatography with thin-layer chromatography, namely F1, F2, F3, and F4.


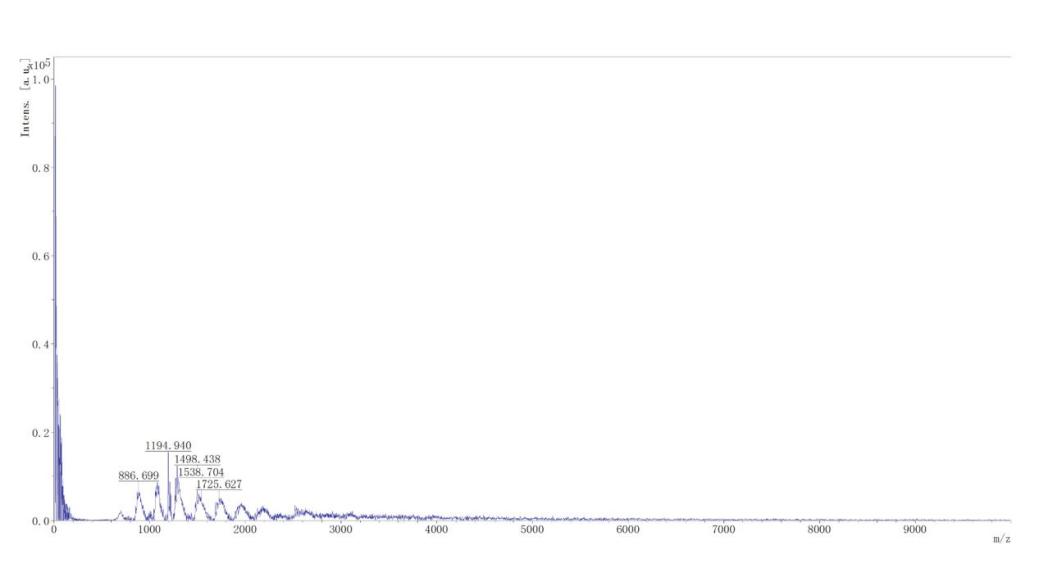


**Supplementary Figure 3.** Explore the specific active substance of strain MTC-8. Metabolite analysis of the F3 extract and CK by using liquid chromatography-mass spectrometry (LC–MS) technique.


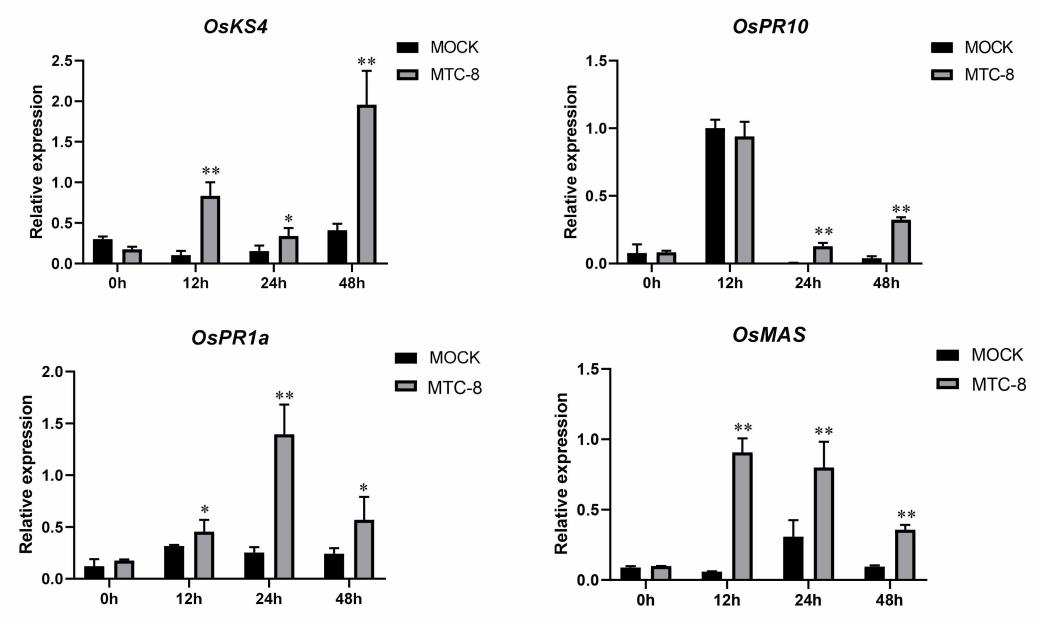


**Supplementary Figure 4.** RNA levels of *OsKS4*, *OsPR10*, *PR1a*, and *OsMAS* genes in rice leaves after sprayed with MTC-8 extracts after 0, 12, 24, and 48 h (sterile water as control). Error bars indicate SD, n = 3 repeats. The values are the means ± SDs. Asterisks denote a significant difference from the MOCK as determined by Student’s t-test (*, 0.01< *p* < 0.05; **, *p* < 0.01).
